# Case Report: A case of necrotizing sarcoid granulomatosis mimicking metastatic tumor

**DOI:** 10.3389/fonc.2025.1703599

**Published:** 2025-11-19

**Authors:** Lingjun Dong, Linhai Fu, Desheng Wei, Guangmao Yu

**Affiliations:** Department of Thoracic Surgery, Shaoxing People’s Hospital, Shaoxing, Zhejiang, China

**Keywords:** lung cancer, necrotizing sarcoid granulomatosis, metastatic tumor, multidisciplinary team, surgical biopsy

## Abstract

This article presents a case study of a patient with early-stage lung cancer who received comprehensive management at our institution throughout the entire clinical course. During follow-up, multiple progressively enlarging solid nodules were detected in the right lung and pleural cavity. Positron emission tomography-computed tomography (PET-CT) demonstrated increased fluorodeoxyglucose (FDG) uptake in these nodules, with a maximum standardized uptake value (SUVmax) of 3.568. Following a multidisciplinary team (MDT) discussion, surgical resection of the nodules was undertaken. Pathological examination confirmed the diagnosis of necrotizing sarcoid granulomatosis (NSG), with special staining and microbiological testing yielding negative results, thereby excluding infectious lesions and tumor metastasis. This case highlights the critical importance of distinguishing metastatic tumors from NSG when new intrapulmonary or pleural nodules appear post-lung cancer surgery. Surgical biopsy is demonstrated to be an effective modality for achieving a definitive diagnosis.

## Introduction

Liebow was the first to describe necrotizing sarcoid granulomatosis (NSG), a rare idiopathic benign lung illness, in 1973. Although its exact etiology is unknown, it is thought to be related to immunological dysregulation, genetic predisposition, and infectious agents (such as fungus and atypical pathogens) (1, 2). According to epidemiological data, NSG primarily affects women between the ages of 20 and 60 (3). Sarcoid-like granulomas, vasculitis, and necrotic lesions are histopathologically indicative of NSG (4, 5). Multiple bilateral pulmonary nodules are the typical radiological manifestation, typically without hilar or mediastinal lymphadenopathy (3, 6). In this paper, we detail an NSG case that was preoperatively suspected to being a lung metastatic tumor. We intend to investigate the variations in imaging characteristics between NSG and pulmonary malignancies, as well as their corresponding treatment approaches, by combining clinical data, diagnostic and therapeutic procedures, and a study of the literature.

## Case presentation

**Figure 1:**
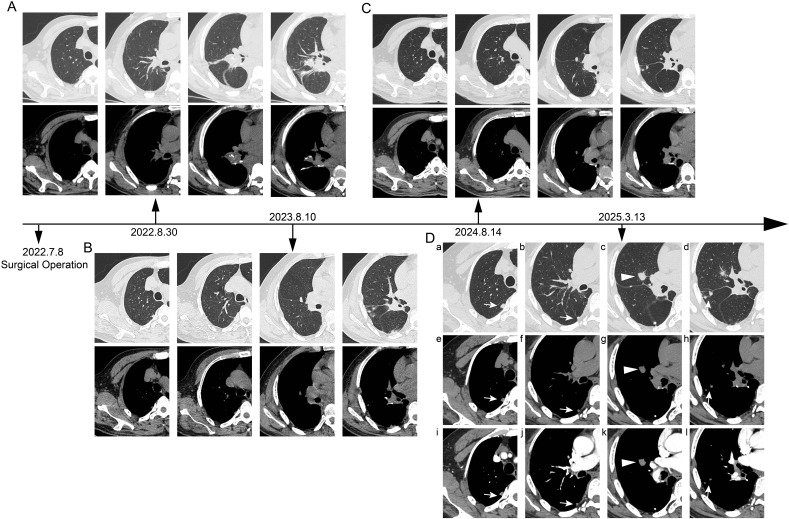
Timeline of postoperative chest CT follow-up. **(A)** Postoperative day 53 (August 30, 2022): Chest computed tomography (CT) scan revealed local atelectasis at the surgical site, with no new subpleural lesions observed. **(B)** 1 year postoperatively (August 10, 2023): Follow-up chest CT demonstrated a solid nodule (approximately 16×13 mm) in the lateral segment of the right middle lobe, along with additional subpleural solid nodules. **(C)** 2 years postoperatively (August 14, 2024): Follow-up chest CT showed no significant interval change in the nodule within the lateral segment of the right middle lobe or the subpleural nodules compared to the previous year. **(D)** Preoperative contrast-enhanced chest CT. On lung window **(a-d)** and mediastinal window **(e-h)** settings, several round nodules (open arrows and arrowheads) are observed in the subpleural region of the right upper lobe and the right middle lobe **(c, g)**. The nodule in the right middle lobe exhibits short spiculations and pleural indentation. **(i-l)** The nodules in the subpleural area of the right upper lobe and the right middle lobe demonstrate contrast enhancement.

A 63-year-old male patient was admitted on March 13, 2025, with a history of right lung cancer surgery performed over 2 years ago and found to have an enlarging right pulmonary mass for over 1 year. The patient had undergone Video-assisted thoracoscopic surgery (VATS) right lower lobe posterior basal segmentectomy in July 2022 for a lung adenocarcinoma, staged as pT1bN0M0, stage IA2. Postoperatively, he was followed up regularly with chest computed tomography (CT) scans. On August 10, 2023, chest CT revealed a 12×6 mm solid pulmonary nodule in the lateral segment of the right middle lobe and several solid nodules in the subpleural area of the right upper lobe posterior segment and at the surgical site. Serial follow-up showed gradual enlargement. Thereafter, an annual chest CT examination was performed on the patient. (**Figure 1**). A CT performed one week before admission demonstrated an enlarged right middle lobe lesion measuring 16×13 mm (mean CT value –15.4 Hounsfield units (Hu)), with well-defined margins, suspicious for malignancy. Multiple solid nodules in the right upper lobe posterior subpleural region and at the surgical site were also noted to have increased in size.

The patient denied cough, sputum, chest tightness, dyspnea, fever, night sweats, or fatigue. Physical examination was unremarkable except for a well-healed surgical scar. Laboratory findings showed elevated alanine aminotransferase (ALT) (65.9 U/L) and gamma-glutamyl transferase (GGT) (152.6 U/L), while C-reactive protein (CRP) and complete blood count were within normal limits.

**Figure 2:**
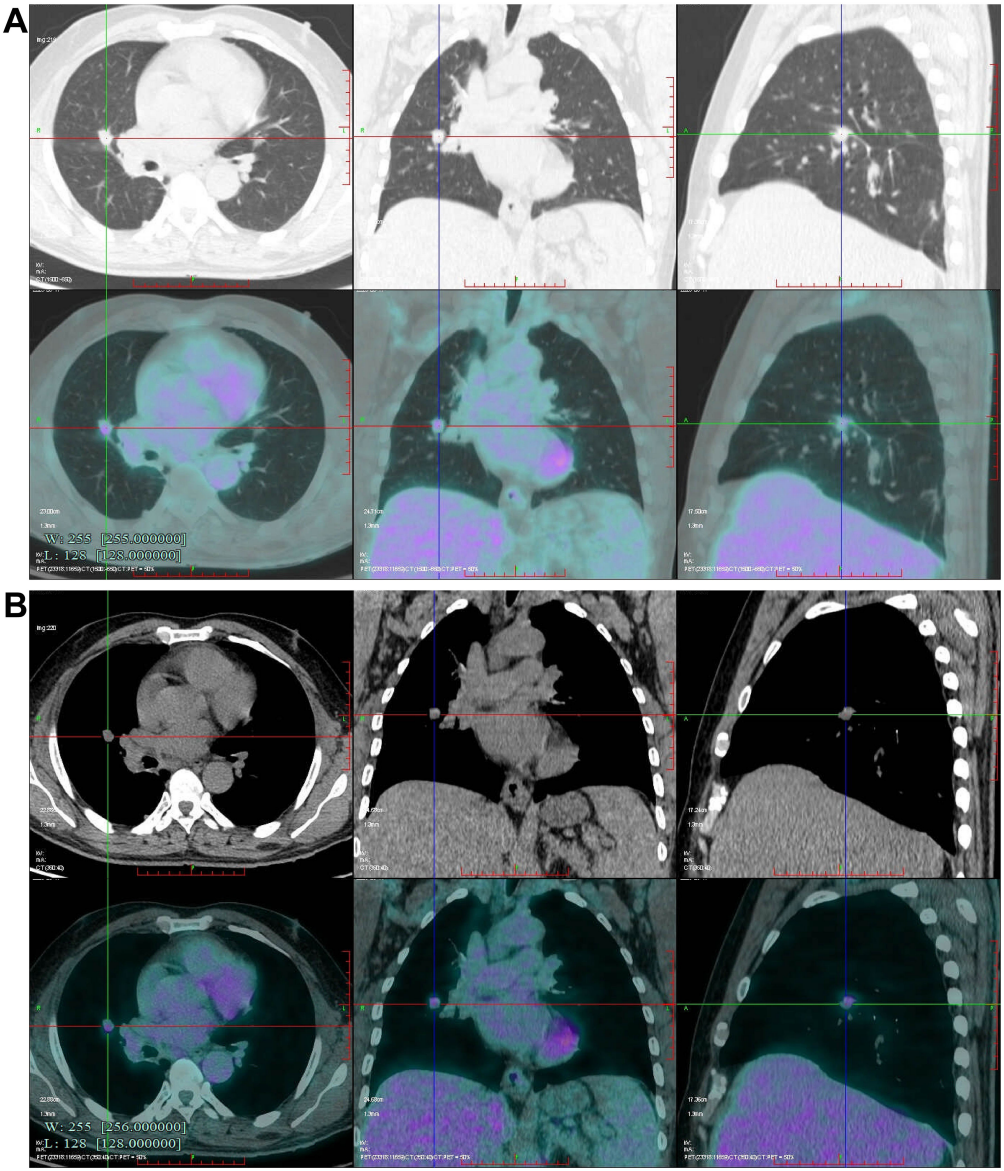
Preoperative Positron emission tomography-computed tomography (PET-CT) scan. **(A, B)** Fluorodeoxyglucose (FDG) uptake (standardized uptake value, SUVmax 3.568) is observed in the nodule located in the right middle lobe.

After admission, a contrast-enhanced chest CT scan revealed multiple solid nodules in the subpleural area of the right upper lobe posterior segment, the lateral segment of the right middle lobe, and the surgical field of the right lower lobe. The findings were categorized as Lung Imaging Reporting and Data System (Lung-RADS) 4A. Postsurgical changes were observed in the right lower lobe, along with multiple small bilateral pulmonary nodules (**Figure 1**). A bone emission computed tomography (ECT) scan showed no evidence of bone metastasis. Tumor markers revealed a prostate antigen ratio of 0.189. CRP, erythrocyte sedimentation rate (ESR), and procalcitonin were within normal limits. Fungal assays, including the (1,3)-β-D-glucan (G) test, galactomannan (GM) test, cryptococcal capsular antigen, and tuberculosis (TB) infection T-cell assays, were all negative. Given the patient’s history of lung cancer, metastatic disease could not be excluded for the right lung lesion. After multidisciplinary team (MDT) discussion, preoperative positron emission tomography–computed tomography (PET-CT) was performed. PET-CT demonstrated a right middle lobe nodule measuring approximately 12 × 11 × 14 mm, with partially well-defined margins and fine spiculation at the periphery. The lesion exhibited increased uptake of fluorodeoxyglucose (FDG), with a maximum standardized uptake value (SUVmax) of 3.568, suggestive of metastatic disease (**Figure 2**). Surgical treatment was therefore undertaken.

**Figure 3:**
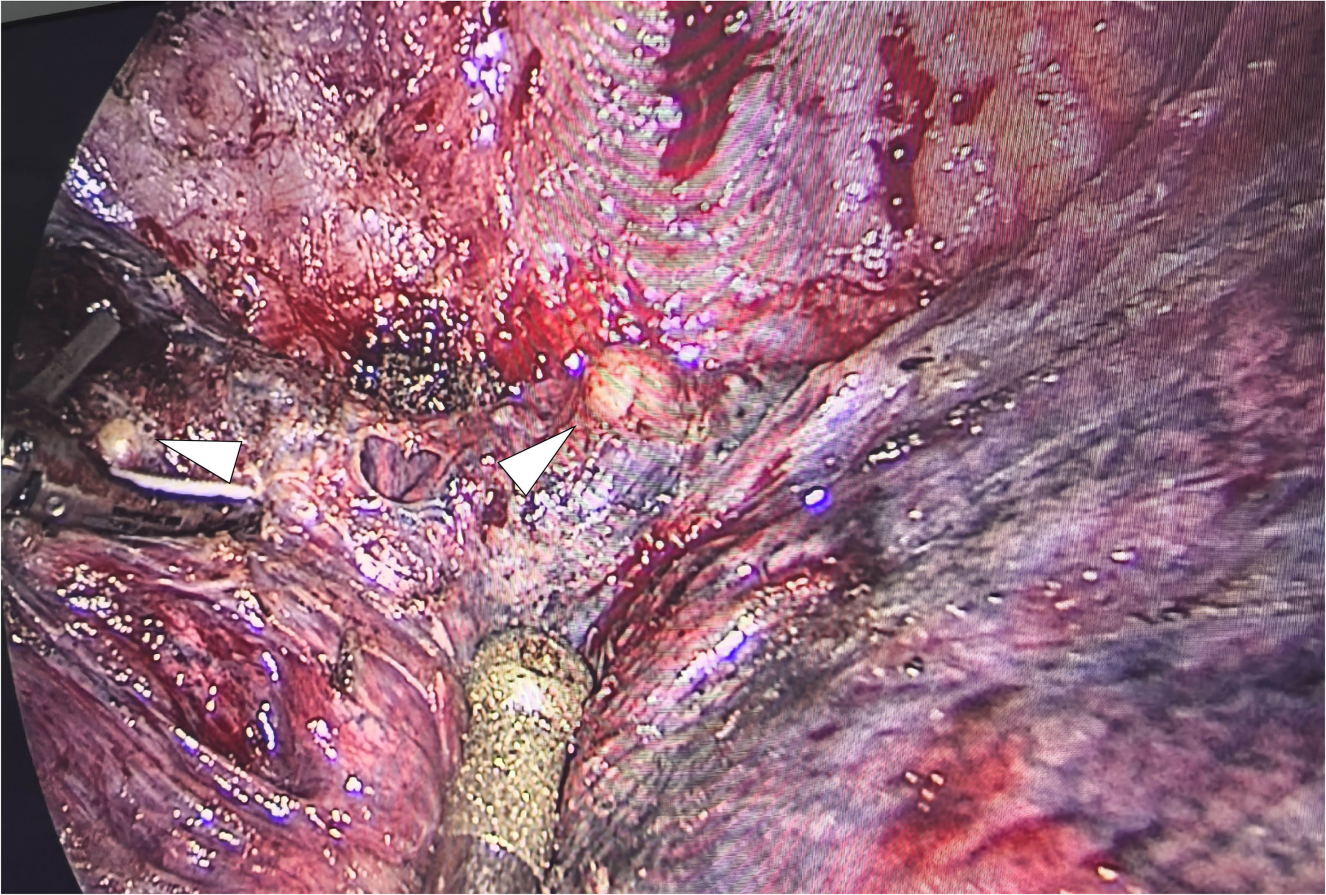
Intraoperative endoscopic gross photograph of the nodule in the right middle lobe. The nodules appear round, pale yellow, and have well-defined margins.

**Figure 4:**
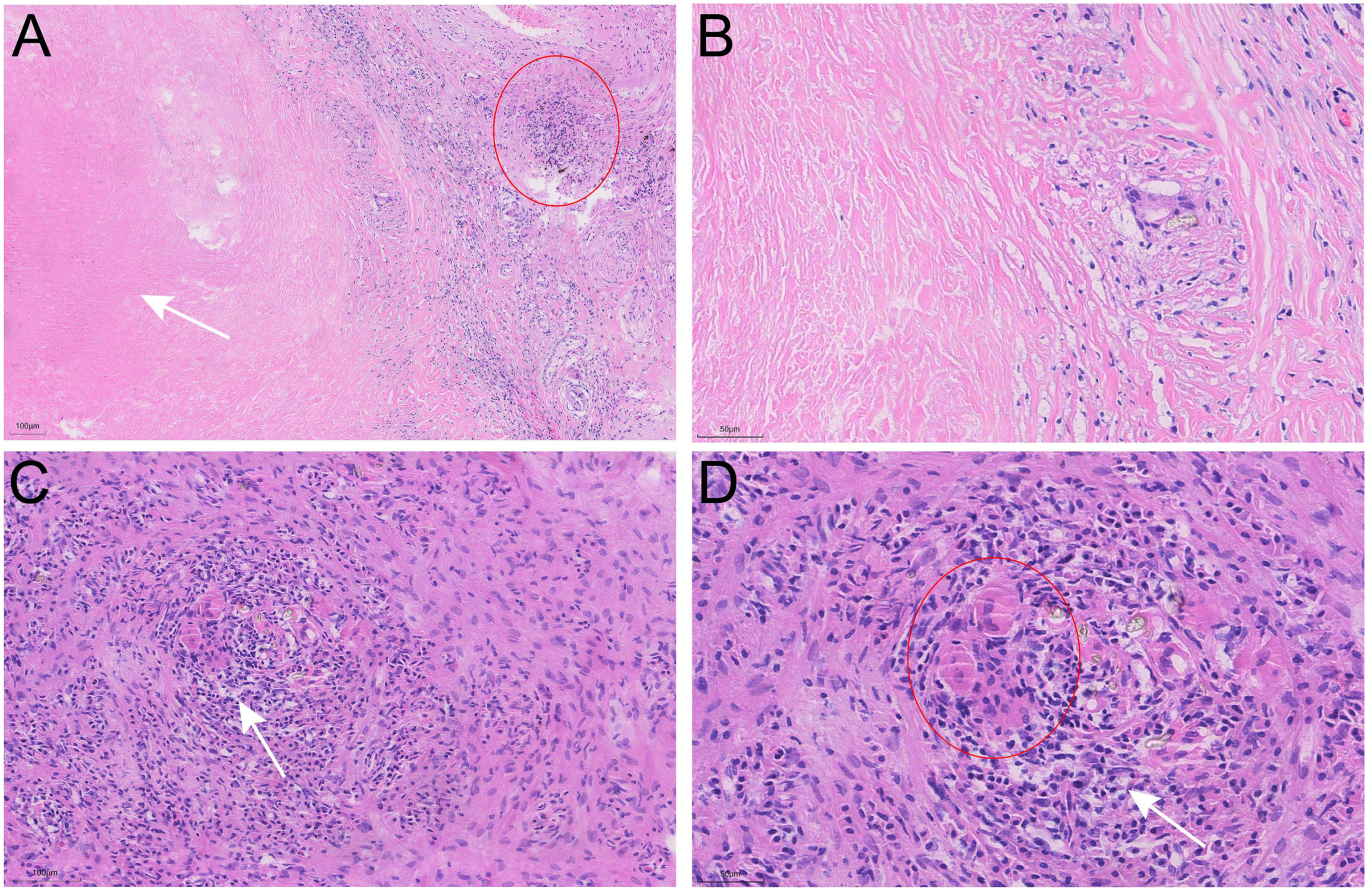
Pathological characteristics of the nodule in the right middle lobe resected by video-assisted thoracoscopic surgery (VATS). **(A, B)** Hematoxylin-Eosin (H&E) staining reveals extensive areas of necrosis (arrows), inflammatory cell infiltration (circle), and nodular granulomas within the nodule. **(C, D)** Multinucleated giant cells (circle) and vasculitis with lymphocytic infiltration (arrows) are observed.

The patient underwent a VATS lung biopsy. After being found to be white in color and firm in consistency during surgery, the nodules in the right upper lobe, right middle lobe, and visceral pleura were removed for biopsy (**Figure 3**). Analysis of frozen sections showed widespread coagulative necrosis and granulomatous inflammation. The final histological analysis verified that the perilesional stroma had intense lymphocytic infiltration and granulomatous inflammation with widespread necrosis (**Figure 4**). The results of special staining, such as acid-fast staining (negative) and periodic acid-Schiff (PAS) staining (negative), were all non-reactive. NSG was the diagnosis supported by the histopathological results.

Given that the patient’s laboratory tests showed no abnormalities and all enlarged nodules had been surgically resected, the attending physician recommended continued medical observation postoperatively, with no initiation of glucocorticoid therapy.

## Discussion

After sublobar resection for stage IA2 early lung cancer, multiple solid nodules in the right lung were found to be progressively enlarged during follow-up. Imaging findings and medical history highly suggest metastasis. However, the pathological findings were confirmed as necrotizing nodular granuloma. Necrotizing nodular granulomatosis is a rare benign disease of the lung that is histologically characterized by necrotizing granulomatosis with vasculitis and massive necrosis and is often clinically confused with pulmonary malignancy, attributed to its highly similar imaging features to peripheral lung cancer, and its size may grow over time, often resulting in misdiagnosis, which may lead to unnecessary systemic treatment (7–10). Similarly, case reports have documented that sarcoidosis can present as a solitary pulmonary nodule or mass, with imaging features closely resembling those of lung cancer, potentially leading to misdiagnosis and unnecessary invasive procedures or treatment (11). Thus, nodules of 8 mm or larger usually require further refinement on PET-CT scan for evaluation. Some scholars believe (12) that PET-CT can effectively distinguish between benign and malignant Solitary pulmonary nodule, and its positive results can be used as the basis for pulmonary resection surgery. However, although FDG PET may be of ancillary value in metabolically inactive lung lesions, the occurrence of false-positive results is a significant problem for granulomatous disease (6, 13). As the PET-CT scan results of this patient suggested elevated FDG uptake in multiple solid nodules in the right lung surgical area (Suvmax 3.568), combined with the history of lung cancer, lung cancer recurrence was highly suspected, so our team did a thoracoscopic lung biopsy.

Since pulmonary necrotizing nodular granuloma is highly similar to lung cancer and there are only a few articles on its radiological features, the clinical diagnosis is difficult. Recent research by Catelli et al. has shed important light on this diagnostic dilemma. Their findings elucidated that in patients with sarcoidosis, the presence of spiculation, a larger diameter, elevated CT attenuation (e.g., >60 HU), and a solitary presentation are distinct radiological characteristics significantly correlated with a higher probability of malignancy (14). A classification system has been developed by combining the clinical baseline characteristics of patients with chest thin-section computed tomography (TSCT) features, it also includes eight types with different characteristics (15). This classification scheme can provide effective guidance for clinical practice to distinguish granulomatous inflammation from lung cancer. In addition, the team retrospectively analyzed the clinical and imaging features of pulmonary granulomatous inflammation and lung cancer through a case-control study. Studies have found that in young patients with diabetes (≤63 years), nonenhanced CT values (≤21hu), irregularly shaped, and non-moderately enhanced SNS should first be suspected as granulomas (16). It is helpful to recognize and understand the clinical and radiological features of necrotizing nodular granulomas to guide the appropriate diagnosis, treatment and management.

Surgical biopsy is a reliable diagnostic tool for new nodules of lung cancer that can not be identified on imaging (9). And compared with other interventional methods such as puncture, surgery can simultaneously explore and biopsy multiple or even all nodules on the same side. Even if puncture is guided by CT, there is also the possibility of false negatives and the inability to deal with smaller nodules. This patient underwent a thoracoscopic procedure to explore all the nodules and obtain sufficient tissue according to the preoperative imaging guidance. Metastatic adenocarcinoma and infectious disease were excluded, unnecessary chemoradiotherapy or targeted therapy is avoided. Therefore, we believe that there is the possibility of coexistence of benign nodules and metastatic lesions of lung cancer in theory. We should try to explore and sample comprehensively during the operation to avoid missed diagnosis of metastases.

## Conclusions

New intrapulmonary/pleural nodules after surgery for early stage lung cancer need to be differentiated between metastatic tumors and non-neoplastic lesions (e.g., NSG), and it is inappropriate to blindly adopt systemic therapy (chemotherapy or targeted therapy, etc.) without the basis of pathologic confirmation, and surgical biopsy is an effective means of clarifying the diagnosis and guiding the treatment.

## Data Availability

The raw data supporting the conclusions of this article will be made available by the authors, without undue reservation.
